# Comparing clinical decision-making between colposcopists and large language models in cervical dysplasia management: a pilot prospective multicenter study

**DOI:** 10.1007/s00404-026-08498-w

**Published:** 2026-06-18

**Authors:** Jan Lennart Stalp, Juliane Alexandra Schneider, Lena Steinkasserer, Jens Hachenberg, Matthias Jentschke, Peter Hillemanns, Dominik Wolff, Agnieszka Denecke

**Affiliations:** 1 Department of Obstetrics and Gynecology, Hannover Medical School, Carl-Neuberg-Str. 1, 30625 Hannover, Germany; 2 Commission Digital Medicine, German Society for Gynecology and Obstetrics (DGGG), Berlin, Germany; 3 Peter L. Reichertz Institute for Medical Informatics (PLRI) of TU Braunschweig and Hannover Medical School, Hannover Medical School, Hannover, Germany; 4 Department of Obstetrics and Gynecology, Klinikum Würzburg Mitte, Würzburg, Germany

**Keywords:** Cervical intraepithelial neoplasia, Large language model, Medical guideline, Clinical decision support system, ChatGPT

## Abstract

**Purpose:**

This prospective multicenter study aimed to compare the decision-making abilities of board-certified colposcopists and two commercially available large language models (LLM), ChatGPT-4o and ChatGPT-5, in cervical dysplasia management.

**Methods:**

Twenty-three anonymized real-life patient cases with multiple-choice (MC) questions regarding treatment decisions were used to assess answer quality. Ten board-certified colposcopists and the two LLMs addressed the MC questions. The gold standard was defined by two guideline authors. LLMs were prompted to justify their responses. Concordance rates were calculated and compared across all questions and histopathological subgroups, including cervical intraepithelial neoplasia (CIN), unspecific histopathological results, and cervical cancer cases.

**Results:**

Clinicians and LLMs achieved similar overall concordance rates compared to the gold standard (69.6% for clinicians, 69.6% for ChatGPT-4o, and 65.2% for ChatGPT-5). ChatGPT-5 outperformed clinicians in precancerous lesions (81.8% vs. 66.4%), while clinicians excelled in complex cases with unspecific histopathology (86% vs. 60%). Clinicians showed a tendency to overtreat low-grade lesions (CIN I), opting for more intensive surveillance. ChatGPT-4o performed better than ChatGPT-5 in cervical cancer cases, though both models struggled with these scenarios.

**Conclusion:**

This study highlights the potential of LLMs as decision support tools in cervical dysplasia management, particularly for straightforward cases like precancerous lesions. However, clinicians remain superior in handling complex or ambiguous cases. The tendency of clinicians to overtreat low-grade lesions may offer the potential to test the implementation of a decision support tool for those cases. While LLMs show promise, exploring open-ended clinical scenarios and integrating retrieval-augmented generation could enhance their practical application.

**Supplementary Information:**

The online version contains supplementary material available at 10.1007/s00404-026-08498-w.

## What does this study add to the clinical work

**Table:** 

This study demonstrates the capabilities of large language models (LLMs) in cervical dysplasia management, particularly in straightforward cases of precancerous lesions. The study highlights the potential of LLMs to support clinicians, while emphasizing the need for machine-readable guidelines and further refinement of LLMs to integrate them effectively into clinical practice.	

## Introduction

Managing cervical dysplasia is a fundamental part of cervical cancer prevention and gynecological routine work. Yearly, around 39.000 patients are seen and treated in certified dysplasia units in Germany [1]. High-quality diagnostics and early treatment might then prevent the occurrence of cervical cancer, ensure patients’ health, and reduce healthcare costs. Hence, treatment decisions are standardized and based on regularly updated guidelines [2, 3]. Nevertheless, in contrast to cancer therapy, all decisions are made without a certified board being involved. To ensure quality standards, colposcopists are certified after finishing specific training courses and an exam to manage these cases. Still, clinical decision-making might vary between practitioners due to several influencing factors [4–6]. To support clinicians, artificial intelligence (AI)-based systems, for instance, in colposcopic image recognition, are the focus of recent developments [7]. In breast cancer screening, double evaluation (AI/clinician) is already improving workflows, diagnostic specificity, and therefore patient safety [8–10]. Further support could be implemented in AI-based clinical decision support for therapeutic recommendations. This has not yet been evaluated in the context of dysplasia management. Previous work proved the outstanding performance of large language models (LLM) in multiple-choice (MC) questions from medical exams, including case-related therapy decisions [11, 12]. Even though real-life patient casuistics might prove to be more complex than medical exam MC questions, the existing potential of broad, commercially available LLM tools is undeniable. These systems can process and analyze natural language input such as medical guidelines, studies, and patient casuistics, and provide the user with a structured treatment recommendation based on the provided input. The capability of LLMs in guideline-based decision-making has been shown previously in early breast cancer [13–16]. Still, limits such as incorrect reasoning or hallucinations require further studies. For this purpose, *Roy* et al*.* developed a classification scheme to further characterize wrong output justifications and allow a better understanding of false reasoning in complex question situations [17]. With the release of the OpenAI Inc. (San Francisco, CA, USA) ChatGPT-5, hope arises for more sufficient and comprehensible recommendations. In cervical dysplasia treatment, the comparability and potential benefits of LLM-supported decision-making have not yet been evaluated. Therefore, we conducted a first-of-its-kind prospective multicenter study comparing treatment recommendations for cervical dysplasia patients by 10 certified colposcopists as well as ChatGPT-4o and ChatGPT-5 on an MC questionnaire. Additionally, the LLM was prompted to justify its output, which was then further classified.

## Methods

### Casuistics, gold standard, and colposcopists

Adapted and anonymized real-life patient cases with cervical intraepithelial neoplasia (CIN), uncertain histopathology, or cervical cancer treated in the Department of Obstetrics and Gynecology of Hannover Medical School were used to prepare 30 casuistics. Each case included a question concerning the treatment recommendation in an MC manner with four answer possibilities. The casuistics included different complexities to cover the spectrum of cervical dysplasia. The patient cases were structured and shortened to therapeutically relevant details (e.g., age, family planning, medical preconditions, and previously performed surgery) and histological classification if available. Information extent varied in width and depth, but was sufficient to answer the MC questions.

The gold standard was defined by two authors of the German AWMF (“*Arbeitsgemeinschaft der Wissenschaftlichen Medizinischen Fachgesellschaften e.V.*”, engl. Working Group of Scientific Medical Societies) cervical cancer prevention guideline to ensure guideline concordance. After this process, seven cases were excluded due to answer variations between the experts (2 cases) or ambiguous cases (5 cases). The final 23 cases, MC questions and correct answers are displayed in Supplemental File 1.

For our prospective multicenter approach, a total of 10 specialized colposcopists certified by the “*Arbeitsgemeinschaft Zervixpathologie und Kolposkopie e.V.*” (AG-CPC, engl.: Board of the German Society of Colposcopy and Cervical Pathology) were involved. Participants are practicing at different-sized facilities (private practice, hospital, university clinics) with several years of clinical experience.

### LLM and prompt engineering

ChatGPT-4o and ChatGPT-5 were accessed via the OpenAI online login and used in default mode. Settings such as temperature or the amount of tokens cannot be modified via the online login and are set based on an internal algorithm triggered by the prompt. A paid license was deployed to guarantee the most recent versions. Casuistics were prompted in groups of 5 on the 20th of July 2025 (ChatGPT-4o) and on the 19th of October 2025 (ChatGPT-5). A new conversation was opened for every input. The prompt was designed to be simple and requested a justification for both the selected answer and the rejected answers, partially following the prompt *Roy *et al*.* [17] used, leading to the following input: *Please answer the multiple-choice questions in the attached Word document and use both attached PDF documents as a basis for your answers. Only one answer per question is permitted. Please justify your answer and explain why the other options are incorrect.* Casuistics and prompts were provided in German to match the guideline language.

### Medical guidelines

The LLMs were provided with the latest versions of the AWMF guidelines on cervical cancer prevention (Version 1.1, March 2020; [2]) and on diagnosis, treatment, and follow-up care for patients with cervical cancer (Version 2.2, March 2022; [3]). Both are written in German language, consisting of text, tables, and diagrams, and are available in PDF format. The documents were uploaded without preprocessing.

### Classification of incorrect LLM output

To gain deeper insights into incorrect LLM answers, these were classified according to the error taxonomy proposed by *Roy *et al. [17]. This scheme was developed based on wrong LLM answers to USMLE (*United States Medical Licensing Examination*) MC questions, and the results yielded the existence of 4 error categories: *reasoning-based error*, *knowledge-based error, reading comprehension error,* and *non-error,* with multiple errors occurring simultaneously.

### Data analysis

Since this was designed as a pilot study, a power analysis was not conducted beforehand. All answers were summarized in an analysis table and compared to the gold standard. The number of correct answers across clinicians was calculated for each question in percentage. The resulting values were averaged for each diagnosis (CIN I with *n* = 3, CIN II with *n* = 4, CIN III with *n* = 4, cervical cancer with *n* = 7, or unspecific pathology with *n* = 5), and across all questions. Due to the limited number of cases and the structure of the data, a statistical analysis could not be performed, so the results were analyzed descriptively. Given the small number of cases in certain subgroups (e.g., CIN I), the analyses are exploratory in nature and should be interpreted with caution.

Cohen’s Kappa [18] was calculated to quantify inter-rater agreement between the two guideline authors. To quantify answer variability, Shannon entropy [19] was calculated for the clinicians’ answers for each question and visualized in a heatmap plot generated using Matplotlib version 3.10.3 and Seaborn version 0.13.2 in Python [20, 21]. To ease interpretability, the resulting values were normalized to values between 0 and 1 using the maximum entropy, as proposed, e.g., by *Wilcox* [22]. High answer variability results in high entropy, whereas entropy is zero if all clinicians choose the same answer option. Further information about the methods can be found in Supplemental File 2.

For each of the casuistics answered incorrectly by ChatGPT, the reasoning for choosing the final (incorrect) answer was further analyzed qualitatively and classified according to the error taxonomy proposed by *Roy *et al. [17].

## Results

### Analysis of clinical answers

From 30 cases, 5 cases were excluded from analysis due to question ambiguity stated by the guideline authors. For the remaining total of 25 cases, the guideline authors agreed in 23 cases. Cohen’s Kappa was calculated to 0.8845, indicating high agreement.

All participating clinicians were board-certified by the AG-CPC and received the casuistics with the MC questions. Answers were collected anonymously and compared to the gold standard.

**Fig. 1:**
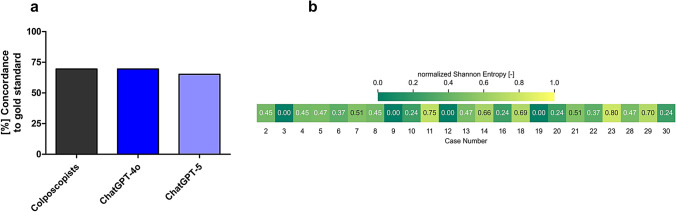
Answer concordance of colposcopists, ChatGPT-4o and ChatGPT-5 with the gold standard and answer entropy among colposcopists. **a** Overall answer concordance (in percent) of certified colposcopists (*n* = 10), ChatGPT-4o and ChatGPT-5 on 23 dysplasia cases with multiple-choice questions and four answer possibilities in comparison to the gold standard. **b** Heatmap of normalized Shannon entropy illustrating the answer variability. Each heatmap value was calculated from the answers of *n* = 10 colposcopists choosing one answer out of four possible answer options. The value “0” (dark green) shows complete homogeneity, whereas “1” (yellow) indicates the maximal heterogeneity

**Fig. 2:**
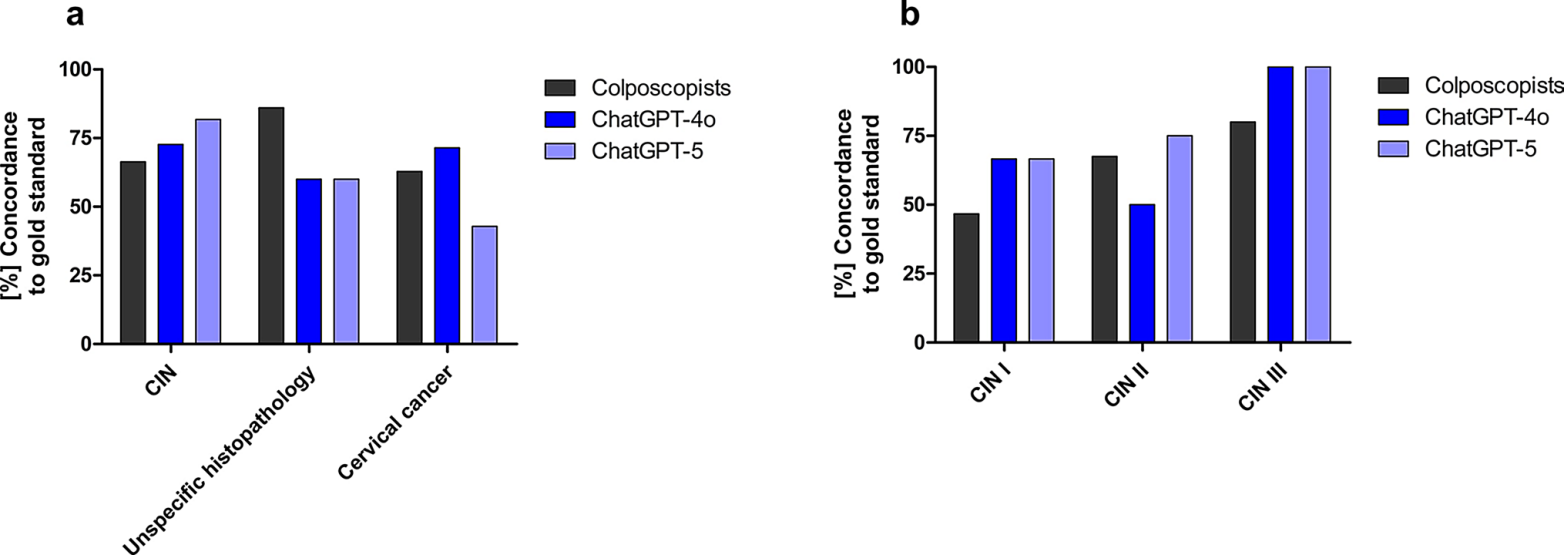
Answer concordance of colposcopists, ChatGPT-4o and ChatGPT-5 by case subgroups compared to the gold standard. **a** Answer concordance (in percent) of certified colposcopists (*n* = 10), ChatGPT-4o and ChatGPT-5 with the gold standard divided in histopathological subgroups CIN (*n* = 11), unspecific histopathology (*n* = 5) and cervical cancer (*n* = 7). **b** Answer concordance (in percent) of certified colposcopists (*n* = 10), ChatGPT-4o and ChatGPT-5 with the gold standard divided in CIN subgroups CIN I (*n* = 3), CIN II (*n* = 4) and CIN III (*n* = 4)

Across all cases, clinicians achieved a mean concordance rate of 69.57% with the gold standard (Fig. 1a). The answer heterogeneity between all clinicians is shown in Fig. 1b, indicating different opinions across 19 of 23 cases (82.6%). After dividing the casuistics into subgroups of histopathological diagnoses, clinicians reached a score of 66.36% for precancerous lesions (Fig. 2a). If separated into the CIN subgroups, the following concordance rates were achieved: 46.67% for CIN I, 67.5% for CIN II, and 80% for CIN III (Fig. 2b), indicating discrepancies in the management of low-grade lesions. In case of unspecific histopathology or cervical cancer casuistics, participating colposcopists reached a concordance rate of 86% and 62.86%, respectively (Fig. 2a).

Analyzing the wrong answers revealed a clinical tendency for the safest treatment option rather than the gold standard option for CIN I. For example, in the case of a 26-year-old patient with transformation zone type I and a histopathological CIN I (case 4), 70% of clinicians favored a second invitation to the dysplasia unit with re-biopsy after 6 months, whereas the gold standard recommended cytological control at the treating gynecologist’s practice.

A comparative qualitative analysis of the colposcopists’ responses to histopathological CIN II cases revealed both overtreatment and undertreatment, without a distinct trend. In contrast, for CIN III cases, the incorrect answers primarily involved undertreatment.

### LLM output and clinical comparison

Overall, ChatGPT-4o and ChatGPT-5 reached similar concordance rates with 69.57% and 65.22%, respectively (Fig. 1a). Interestingly, the older version was capable of slightly outperforming the newer model. When analyzing the subgroups, ChatGPT-4o achieved a concordance rate to the gold standard of 72.73% for CIN, 60% for unspecific histopathology, and 71.43% for cervical cancer casuistics (Fig. 2a). Within the precancerous lesions, ChatGPT-4o reached concordance rates of 66.67% for CIN I, 50% for CIN II and 100% for CIN III cases (Fig. 2b).

In comparison, ChatGPT-5 achieved concordance rates of 81.82% for CIN, 60% for unspecific histopathology, and 42.86% for cervical cancer cases (Fig. 2a). When analyzing the CIN subgroups, ChatGPT-5 reached concordance rates of 66.67% for CIN I, 75% for CIN II, and 100% for CIN III casuistics (Fig. 2b). Consequently, the newer model exhibited superior performance in addressing the treatment of precancerous lesions, overall and for the CIN II subgroup. However, the older model demonstrated superiority in the management of cervical cancer cases.

In contrast to clinical answers, both ChatGPT versions showed greater concordance with the gold standard for precancerous lesions, with ChatGPT-5 achieving the highest results, while clinicians proved superiority for challenging cases with unspecific histopathology. Interestingly, for cervical cancer cases, ChatGPT-4o as well as the clinicians reached higher concordance rates than the ChatGPT-5 model.

Regarding case 21 with cervical cancer, both models referenced the recently published SHAPE study [23], not yet implemented in the German guideline, but already into clinical routine. Interestingly, ChatGPT-4o used the study results and chose the correct answer, demonstrating the use of sources outside the guideline and a failure to adhere to the specified prompt. ChatGPT-5 also referred to the SHAPE study but did not utilize the results, stating that the study “…*has not yet been fully implemented; currently not standard*…”. In summary, this proved the greater capabilities of ChatGPT-5 to follow a given prompt and the model’s superior reasoning in this case, despite choosing the clinically incorrect answer.

### Analysis of incorrect LLM output

**Fig. 3:**
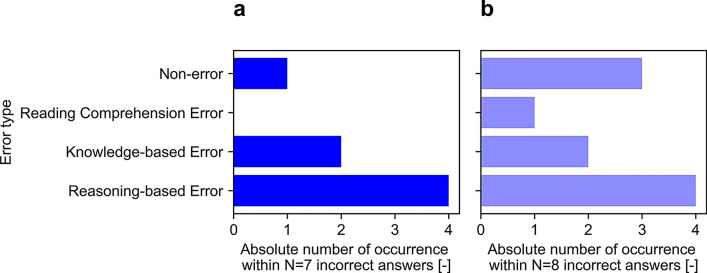
Distribution of top-level error types in incorrect answers of ChatGPT. Error types refer to the error taxonomy proposed by *Roy *et al. [17]. Multiple errors per incorrect answer were possible. **a** Results of ChatGPT-4o. **b** Results of ChatGPT-5

In contrast *to Roy *et al. [17], the prompt in our study explicitly instructed the models to use the relevant guidelines that were uploaded simultaneously. Interestingly, in some cases, the answer parts could not be clearly assigned to one of the seven error types. Therefore, the upper taxonomy level proposed by *Roy *et al*.*, consisting of four top-level error categories: *non-error type, reasoning-based error, knowledge-based error,* and *reading comprehension error*, was applied [17]. For ChatGPT-4o, within a total of seven incorrect answers, seven errors were identified, of which one was classified as a *non-error type*, four as *reasoning-based errors*, and two as *knowledge-based errors* (Fig. 3a). For ChatGPT-5, within the total of eight incorrect answers, ten errors were classified, of which three belonged to a *non-error type*, four to *reasoning-based errors*, two to *knowledge-based errors*, and one to *reading comprehension error* (Fig. 3b).

All *non-error type* errors belonged to the subtype *non-error type 1 (reasonable response),* where the models gave a reasonable answer despite choosing the incorrect answer option*.* Reasons for this error type were, e.g., recent study updates (case 21; ChatGPT-5) or interpretable guideline scope (case 8; both models). Detailed classification results can be found in Supplemental File 3.

## Discussion

In this study, we conducted a first-of-its-kind prospective multicenter analysis comparing clinical decision-making of ten board-certified colposcopists with two commercially available LLMs (ChatGPT-4o and ChatGPT-5) in cervical dysplasia management. Overall, our results showed similar concordance rates to the gold standard with higher answer concordance by ChatGPT-5 for precancerous lesions, whereas clinicians outperformed the LLMs in complex cases.

Even though clinical decision-making regularly follows national and international guidelines, differences can arise between clinicians due to varying contextual factors or therapeutic preferences [5]. In cervical dysplasia management, treatment decisions are based on the patient’s history, a Papanicolaou (PAP) smear, human papillomavirus (HPV) testing, colposcopy, and, most importantly, a histopathological sample diagnosing a precancerous lesion and altogether estimating the risk of developing cervical cancer. As spontaneous regression rates are high, up to 90%, for low-grade squamous intraepithelial lesions, active surveillance is frequently the chosen treatment option [24]. Nevertheless, the answer concordance rate within the CIN I subgroup by the participating clinicians was the lowest for all precancerous lesions. The given answers mainly included overtreatments such as short-term re-colposcopy or re-biopsy. Even though a re-visit might not harm the patient, relevant additional healthcare costs arise consequently within a country, estimated for the United States of America at several billion dollars per year [25]. A survey interviewing clinicians about overtreatment by *Lyu *et al*.* revealed that practitioners estimate around 20.6% of the overall medical care as unnecessary, with the leading reasons being fear of malpractice and patient pressure/request [26]. Therefore, solutions are needed to support clinicians with their choice of treatment and reduce the risk of potentially unnecessary visits or diagnostics. Since board decisions regarding oncological patient care are impractical to implement into daily dysplasia management, an LLM-based decision support tool might offer additional guidance in those cases.

Nevertheless, indicating a treatment decision can be a highly patient-specific task with several influencing factors. Still, recent publications indicate the capabilities of LLM decision support tools, for instance, in emergency internal medicine cases [27]. Challenges with commercially available LLMs arise if treatment decisions are verified for guideline compliance or if the input prompt demands a literature reference, as those models are vulnerable to hallucinations [28, 29]. Our overall results showed that the deployed LLMs are equivalently capable of recommending an MC question-based guideline-compliant treatment in cervical dysplasia management compared to board-certified colposcopists, but with relevant differences. Since ChatGPT-5 outperformed the clinicians for precancerous lesions, those cases could serve as a future use-case for clinical implementation of an LLM-based decision support tool. Even though no statistical tests were applied, we considered the higher concordance rate as clinically relevant. Still, given the small sample size, these results must be considered exploratory. For other gynecological pathologies, e.g., breast cancer, similar conclusions were drawn for LLM decision support tools, and focusing on early breast cancer cases for initial proof-of-concept studies was suggested [13]. In comparison, the data confirmed the high relevance of board-certified colposcopists for complex cases with unspecific histopathological results due to the superior concordance rate. Regarding the cervical cancer cases, the concordance rates should be viewed as exploratory because colposcopists are not necessarily trained oncologists and may not serve as the optimal test cohort. Additionally, due to the outdated German cervical cancer guideline and existing new clinical trials (e.g., SHAPE), it is expected that clinical practice will differ from the guideline to provide up-to-date treatment [23]. This circumstance is reflected in the conflicting reasoning of the LLM versions. ChatGPT-4o recommended the treatment because of the existing SHAPE publication, but ignored the prompt. ChatGPT-5, on the other hand, advised against it since it is not yet part of the provided guideline. To overcome this conflict, an updated, machine-readable guideline is needed for follow-up projects. While the AWMF cervical cancer prevention guideline (2020–2022) predates the formalized 2023 risk-based management framework of the American Society for Colposcopy and Cervical Pathology (ASCCP) [30], several of its core elements—such as HPV-centered management, persistence-based triage, and extended follow-up after high-grade lesions—already reflect a risk-adapted clinical rationale. However, in contrast to the ASCCP approach, these strategies are not anchored in quantitatively defined risk thresholds but rely on algorithm-based decision pathways. This temporal and methodological difference should be acknowledged when interpreting our findings. For clinical practice, this implies that management according to AWMF guidelines remains broadly aligned with risk-adapted care but may offer less granularity in risk stratification. In comparison, the ASCCP framework enables more individualized decision-making based on explicit risk estimates, which may be particularly relevant in borderline constellations or complex follow-up scenarios.

Nevertheless, the advantages of bounding an LLM to a certain knowledge base also come with limitations: Edge cases may not be incorporated in the guideline, e.g., therapy for patients of high age with comorbidities. These complex cases require the knowledge of an experienced clinician who can weigh up the patient-specific risks and benefits, as these case details go beyond the guideline’s scope.

Nevertheless, to allow meaningful utilization of such tools, future projects are needed to evaluate the performance in an open question manner to better reflect clinical practice. Furthermore, cloud-hosted LLMs are easy to access for initial studies but certainly lack the required data safety to enable daily usage with real-life patient data. Moreover, models are usually trained on vast amounts of data, which is not necessarily specific to use cases in daily clinical routine. Specific documents like guidelines must be accessed for answering, and citation of relevant text parts is crucial for reliable results. For this purpose, retrieval-augmented generation (RAG) [31] enhanced LLMs are recently attracting attention as this technique can reduce hallucinations, can be deployed locally in compliance with data safety requirements, and with a tailored database (e.g., medical guidelines). Using this method, *Tung *et al*.* developed a RAG-enhanced LLM system for prostate cancer screening that provided a guideline-compliant answer in 95.5% of the 220 cases, outperforming junior clinicians by a large margin [32].

*Reasoning-based error* was the major error type in both models’ answers. This finding is in line with the results of *Roy *et al*.* and *Liévin *et al*.* [17, 33]. Challenges in assigning proper classes mainly arose from the fact that the LLMs were instructed to use two specific guidelines. Thus, a statement not supported by the guideline could be classified either as an *unsupported medical claim* (*knowledge-based error*) or a *hallucination of information* (*reading comprehension error)*. Nevertheless, we kept our classification tight to the taxonomy description and categorized these errors as *knowledge-based errors* and used the *hallucination of information error* for reading comprehension failures concerning the presented casuistic. This highlights the need for a taxonomy suitable for open-book settings like in our study or in RAG-based systems. To provide more insights into the failure origins in the processing pipeline, RAG could be used.

In conclusion, this pilot study demonstrates that both board-certified colposcopists and LLMs like ChatGPT-4o and ChatGPT-5 achieve similar overall concordance rates with the gold standard in cervical dysplasia management. While LLMs excelled over the clinicians in precancerous lesions, clinicians outperformed LLMs in complex cases with unspecific histopathology. The tendency of clinicians to overtreat low-grade lesions underscores the potential for decision support tools to reduce unnecessary healthcare costs. Still, considering the small sample size, a sufficiently powered follow-up study is needed for verification. Due to the chosen test cohort, the results for cervical cancer cases should be viewed as preliminary. LLMs, while effective, require updated guidelines and enhanced data safety for practical applications. Future research should explore their use in open clinical scenarios and integrate retrieval-augmented generation to minimize errors. These findings suggest a promising role for LLMs in supporting clinicians, particularly in straightforward cases, while emphasizing the need for further refinement.

## Supplementary Information

Below is the link to the electronic supplementary material.

Supplementary file1 (PDF 427 KB)

Supplementary file2 (PDF 205 KB)

Supplementary file3 (PDF 4246 KB)

## Data Availability

The original contributions presented in the study are included in the article/ supplementary material; further inquiries can be directed to the corresponding author.
