# Supplementary material for: Comparing clinical decision-making between colposcopists and large language models in cervical dysplasia management: a pilot prospective multicenter study
Source: Arch Gynecol Obstet. 2026 Jun 18;313(1):206. doi: 10.1007/s00404-026-08498-w (PMC13279379; doi:10.1007/s00404-026-08498-w)
Supplement: Supplementary file 1 — Supplementary file1 (PDF 427 KB) [file 404_2026_8498_MOESM1_ESM.pdf]

LLM Cervix Multiple Choice:

Case 2:

40-year-old female patient IIIG. IIIP., family planning complete, no vaccination, HPV positive, cytology Pap III D2 and colposcopy: Type I TZ, major ectocervical changes. Histologically confirmed CIN 2 in the sample

What would be the next steps?

1. Routine check-up with gynecologist in 12 months (cytology and HPV testing) ☐
2. Cytological check-up in 3 months, repeat colposcopy and sampling with referral to dysplasia clinic ☐
3. Surgical treatment in the form of loop excision ☒
4. HSK and fractional abrasion ☐

Case 3:

60-year-old female patient IIIG. IIIP., family planning complete, no vaccination, HPV-positive, cytology Pap III D2 and colposcopy: Type III TZ, major endocervical changes. Histologically confirmed CIN 2, p16 positive in the sample.

What would be the next steps?

1. Routine check-up with gynecologist in 12 months (cytology and HPV testing) ☐
2. Checkup in 3 months, repeat colposcopy and sampling with referral to dysplasia clinic ☐
3. Surgical treatment in the form of loop excision and cervical abrasion ☒
4. Hysteroscopy and fractional abrasion ☐

Case 4:

26-year-old female patient, 0G, 0P. History of HPV vaccination at age 11, with HPV-OT positivity, cytology Pap III D1 and colposcopy: Type I TZ, minor changes. Histologically confirmed CIN 1 in the sample.

What would be the next steps?

1. Routine check-up with the gynecologist in 12 months (cytology and HPV testing) ☒
2. Checkup in 6 months, repeat colposcopy and biopsy with consultation with dysplasia specialist ☐
3. Checkup in 3 months with colposcopy and sample collection, including dysplasia consultation ☐
4. Surgical treatment in the form of loop excision ☐

Case 5:

29-year-old female patient I G. 0 P. Z.n. HPV vaccination at age 17, HPV-positive, cytology Pap IV a-p and colposcopy: Type I TZ, major changes. Histologically confirmed CIN 3 in the sample

What would be the next steps?

1. Routine check-up with gynecologist in 12 months (cytology and HPV testing) ☐
2. Check-up in 3 months, repeat colposcopy and sampling possible with referral to dysplasia clinic ☐
3. Indication for surgical treatment in the form of laser vaporization ☐
4. Surgical treatment in the form of loop excision ☒

Case 6:

35-year-old female patient I G. 0 P. with desire to have children, history of HPV vaccination at age 17, HPV-positive, cytology Pap III D2 and colposcopy: Type I TZ, major changes. Histologically confirmed CIN 3 in the sample, p 16 positive

What would be the next steps?

1. Routine check-up with gynecologist in 12 months (cytology and HPV testing) ☐
2. Checkup in 3 months, repeat colposcopy and sampling with referral to dysplasia clinic ☐
3. Repeat HPV testing with subtyping, cytological check-up in 6 months at the gynecologist ☐
4. Surgical treatment in the form of loop excision ☒

Case 7:

55-year-old female patient IIG. IIP. HPV-positive, cytology Pap IV a-p and colposcopy: Type III TZ. No evaluable material in the sample.

What would be the next steps?

1. Routine check-up with gynecologist in 12 months (cytology and HPV testing) ☐
2. Check-up in 3 months, repeat colposcopy and attempt to take another sample, involving the dysplasia clinic ☐
3. Surgical treatment in the form of cervical dilation, HSK, and fractional abrasion ☐
4. Surgical treatment in the form of loop excision ☒

Case 8:

38-year-old female patient II G. IIP. with completed family planning, no HPV vaccination, smoker with HPV positivity, cytology Pap IV a-g and colposcopy: Type II TZ, minor endocervical changes.

Histologically proven adenocarcinoma in situ in the sample.

What would be the next steps?

1. Routine check-up with gynecologist in 12 months (cytology and HPV testing) ☐
2. Checkup in 3 months, repeat colposcopy and repeat sampling with consultation at the dysplasia clinic ☐
3. Indication for surgical treatment in the form of a hysterectomy ☐
4. Surgical treatment in the form of loop excision ☒

Case 9:

51-year-old female patient, 0 G. 0 P. No vaccination, HPV-16 positive, cytology Pap III p and colposcopy: Type III TZ, major changes. Histologically confirmed CIN 3 endocervical in the sample.

What would be the next steps?

1. Routine check-up with gynecologist in 12 months (cytology and HPV testing) ☐
2. Checkup in 3 months, repeat colposcopy and sampling with referral to dysplasia clinic ☐
3. Repeat HPV testing with subtyping, cytological check-up in 6 months at the gynecologist ☐
4. Surgical treatment in the form of loop excision ☒

Case 10:

32-year-old female patient IG. 0P. with desire to have children, no vaccination, HPV negative, cytology Pap IIp and colposcopy: Type I TZ, minor changes. Histologically proven cylindrical epithelial metaplasia in the sample.

What would be the next steps?

1. Routine check-up with gynecologist in 12 months (cytology and HPV testing) ☒
2. Check-up in 6 months, repeat colposcopy and sampling with referral to dysplasia clinic ☐
3. Checkup in 3 years as part of cervical cancer screening, no further treatment required ☐
4. Further surgical investigation using LEEP and curettage ☐

Case 11:

39-year-old female patient IIG. IIP. with desire to have children, history of HPV vaccination at age 15, HPV-OT positive, cytology Pap II p and colposcopy: Type I TZ, minor changes. Histologically confirmed CIN 1 in the sample.

What would be the next steps?

1. Routine check-up with gynecologist in 12 months (cytology and HPV testing) ☐
2. Checkup in 6 months, repeat colposcopy and sampling with referral to dysplasia clinic ☐
3. Cytological check-up in 6 months at the gynecologist ☒
4. Surgical treatment in the form of loop excision ☐

Case 12:

61-year-old female patient, 0G, 0P, HPV negative, cytology Pap IV a-p and colposcopy: Type III TZ, no sample collection possible due to stenosis.

What would be the next steps?

1. Routine check-up with gynecologist in 3 months (cytology and HPV testing) ☐
2. Checkup in 3 years, repeat colposcopy and sample collection with referral to dysplasia clinic ☐
3. No further treatment due to HPV negativity ☐
4. Surgical treatment in the form of cervical dilation, HSK, and fractional abrasion to rule out other malignancies, if necessary ☒

Case 13:

39-year-old female patient, IVG. IIP., no vaccination, HPV-OT positive, cytology Pap IIp and colposcopy: Type II TZ, major changes. Histologically confirmed CIN 1 in the sample

What would be the next steps?

1. Routine check-up with gynecologist in 12 months (cytology and HPV testing) ☐
2. Checkup in 3 months, repeat colposcopy and sampling with referral to dysplasia clinic ☐
3. Due to discrepancy between major changes on colposcopy and slightly abnormal cytology smear, repeat appointment in 6-9 months for colposcopy and, if necessary, repeat biopsy ☒
4. Surgical treatment in the form of loop excision ☐

Case 14:

43-year-old female patient IIG. IIP., family planning complete, no vaccination, HPV-16 positive, cytology Pap III D2, history of 2 x e conization, and colposcopically: Type III TZ, major changes endocervical. Histologically confirmed CIN 3 in the sample, p16 positive, R1 endocervical protruding into the cervical glands of the uterus.

What would be the next steps?

1. Routine check-up with gynecologist in 6 and 18 months (cytology and HPV testing) ☐
2. Check-up in 3 months, repeat colposcopy and sampling with referral to dysplasia clinic ☐
3. Repeat surgical treatment in the form of loop conization would be possible ☐
4. The indication for hysterectomy ☒

Case 16:

61-year-old female patient IIIG. IIIP., HPV-positive (type unknown), immunosuppression, cytology Pap III p and colposcopy: type II TZ, major changes. Histologically confirmed CIN 2 in the sample

What would be the next steps?

1. Routine check-up with gynecologist in 12 months (cytology and HPV testing) ☐
2. Checkup in 3 months, repeat colposcopy and sampling with referral to dysplasia clinic ☐
3. Repeat HPV testing with subtyping, cytological check-up in 6 months at the gynecologist ☐
4. Surgical treatment as loop excision ☒

Case 18:

53-year-old female patient IVG. IIP. Z.n. LASH due to uterine myomatosis and currently HPV-OT positive, cytology: Pap III D2, colposcopy: Type III TZ, minor endocervical changes visible, biopsy: CIN 2, p 16 positive.

What would be the next steps?

1. Routine check-up with gynecologist in 12 months (cytology and HPV testing) ☐
2. Repeat cytological check in 3 months and, if necessary, repeat biopsy ☐
3. Indication for surgical treatment in the form of conization ☒
4. Indication for surgical treatment as cervical stump extirpation ☐

Case 19:

37-year-old female patient IIG. IP. with desire to have children, history of microinvasive cervical carcinoma, squamous cell carcinoma, stage pT1a, pNx, L0, V0, R0, history of conization, R0 resection, currently under observation after 4 months: Pap III D2, HPV OT positive, colposcopic. Type II TZ, no major changes visible, biopsy: no dysplasia, no evidence of carcinoma.

What would be the next steps?

1. Routine check-up in 3 months with cytology and HPV sampling ☐
2. No action required at present, repeat check-up with cytology and HPV testing in 6 and 18 months after the procedure, hysterectomy after family planning is complete ☒
3. Indication for repeat conization ☐
4. Indication for trachelectomy ☐

Case 20:

38-year-old IIIG. IIP. in the 16th week of pregnancy with Pap IV a- p and HPV 16 positivity. No vaccination. Colposcopic findings: decidualization, large type I TZ, major changes, no atypical vessels, no evidence of invasion.

What would be the next steps in this case?

1. Routine check-up in 3 months with repeat cytology and HPV testing by the gynecologist, further preventive care until delivery, then referral to the dysplasia clinic. ☐
2. Further routine check-ups, HPV vaccination during pregnancy, check-up and biopsy after the 24th week of pregnancy ☐
3. Immediate referral to dysplasia clinic and biopsy ☒
4. Indication for conization during pregnancy due to Pap IV a-p and colposcopic major changes to reduce the risk of bleeding if necessary ☐

Case 21:

44 years old, IIG IIP, history of 2 cesarean sections, most recently 8 months ago, patient is breastfeeding, family planning complete, internally healthy, FA: Brother with pre-leukemia, history of recurrent Pap III D1 and III D2 during pregnancy, HPV 16 positive, colposcopic tumor approx. 2 cm in size in the area of the posterior MM lip, partially located endocervically, PEC of the cervix in the sample, G2. After conization, stage: pT1b1 (1.5 cm diameter, 0.8 cm infiltration depth), pNx, G2, L0, V0, Pn0, R0 (circumferentially ectocervical and endocervical), FIGO IB1

What would be the next steps in this case?

1. Radical hysterectomy according to Wertheim, sentinel lymph node dissection (SLNE) on both sides of the pelvis, possible inclusion in the G-LACC study ☐
2. Analogous to the Shape study: TLH and SLNE pelvic bilaterally. ☒
3. Radical hysterectomy according to Wertheim and complete pelvic and paraaortic lymph node dissection (LNE) ☐
4. Primary chemoradiotherapy ☐

Case 22:

34 years old, 0G 0P, family planning not complete, internally healthy, no HPV vaccination, FA: Mother with cervical carcinoma, history of recurrent Pap III D2, HPV 18 positive, risk factor: contraception, smoking: 5 cigarettes/day, colposcopic approx. 1 cm tumor in the area of the anterior MM lip, completely ectocervical, in the sample PEC of the cervix, G2.
After conization: Cervical carcinoma pT1b1 (0.9 cm diameter, 0.4 cm infiltration depth), pNx, G2, L0, V0, Pn0, R0 (circumferential ectocervical and endocervical), FIGO Ib1

What would be the next steps in this case?

1. Radical hysterectomy according to Wertheim, without oophorectomy, SLNE pelvin bds., possible inclusion in the G-LACC study ☐
2. Analogous to the Shape study: TLH with salpingectomy and SLNE pelvin bds. ☐
3. Trachelectomy and SLNE bds. possible, after completion of family planning Recommendation for simple hysterectomy and salpingectomy bds. ☒
4. Primary chemoradiotherapy ☐

Case 23:

54 years old, 5G 2P, history of 2 cesarean sections, family planning complete, internal medicine: CHD, renal insufficiency II°, history of thrombosis 2 months ago, FA: Brother pancreatic cancer, no regular screening, HPV 16 positive, colposcopic approx. 5 cm tumor in the area of the posterior MM lip, partially located endocervically, infiltration of the vagina visible, no evidence of filiae on CT, no lymphadenopathy visible, PEC of the cervix in the sample, G3. At least FIGO IIA due to vaginal infiltration.
After conization, stage: pT2 (3.2 cm diameter, 2.4 cm infiltration depth), pNx, G3, L1, V0, Pn0, R1 (circumferential ectocervical and endocervical)

What would be the next steps in this case?

1. Radical hysterectomy according to Wertheim with bilateral adnexectomy, bilateral pelvic SLNE, pelvic and para-aortic LNE if necessary, possible inclusion in the G-LACC study ☐
2. Radical hysterectomy according to Wertheim and complete pelvic and para-aortic SLNE ☐
3. Inoperable stage, primary radiochemotherapy taking comorbidities into account without prior surgical staging to prevent surgical comorbidities ☒
4. LSK with pelvic and para-aortic LNE followed by primary chemoradiotherapy ☐

Case 28:

63 years old, 2G 2P, history of 1 cesarean section, internal medicine: healthy, gynecology: no gynecological malignancies, no regular screening, HPV 16 positive, colposcopy shows tumor approx. 6.5 cm in size in the area of the posterior MM lip, partially located in the endocervix, no infiltration malignancies, no regular screening, HPV 16 positive, colposcopic approx. 6.5 cm tumor in the area of the posterior MM lip, partially located endocervically, no infiltration of the vagina, CT scan indicates solitary metastasis in the lung area, in the sample PEC of the cervix, G3.

What would be the next steps in this case?

1. Histological confirmation, PDL-1 status determination, then for CPS<1: cisplatin/paclitaxel/bevacizumab, for CPS>1: cisplatin/paclitaxel/pembrolizumab ☒
2. Primary surgery as Wertheim operation and excision of solitary metastases, followed by adjuvant therapy as combined radiochemotherapy ☐
3. Primary chemoradiotherapy taking comorbidities into account, followed by surgical therapy ☐
4. LSK with pelvic and para-aortic LNE, followed by primary chemoradiotherapy ☐

Case 29:

85 years old, 1G 1P, internal medicine: art. hypertension, CHD, renal insufficiency II°, history of heart attack and stent placement 3 years ago, Crohn's disease, FA: no gynecological malignancies, no regular screening, HPV 16 positive, colposcopic tumor approx. 6.5 cm in size in the area of the posterior MM lip, no infiltration of the vagina, CT scan shows approx. 6.5 cm tumor in the area of the posterior MM lip, no infiltration of the vagina, no regular screening, HPV 16 positive, colposcopic tumor approx. 6.5 cm in size in the area of the posterior MM lip, no infiltration of the vagina, no regular screening, HPV 16 positive, colposcopic tumor approx. 6.5 cm in size in the area of the posterior MM lip, Malignancies, no regular screening, HPV 16 positive, colposcopic approx. 6.5 cm tumor in the area of the posterior MM lip, no infiltration of the vagina, CT scan indicates solitary metastasis in the lung area, no indication of LK-MTS. In the sample collection, PEC of the cervix, G3.
What would be the next steps in this case?

1. Histological confirmation, PDL-1 status determination, followed by CPS<1 cisplatin/paclitaxel/bevacizumab for CPS>1: cisplatin/paclitaxel/pembrolizumab ☐
2. Primary surgery as Wertheim procedure and excision of solitary metastases, followed by adjuvant therapy as radiation ☐
3. Primary chemoradiotherapy taking comorbidities into account, followed by surgical therapy ☐
4. Radiation alone due to age and comorbidities ☒

Case 30:

35 years old, 0G 0P, wants children, medically healthy, HPV 16 positive, regular check-ups, major changes on colposcopy. Incidental finding on PE: PEC, G2.
After conization: PEC of the cervix, 11 mm, infiltration 7 mm, stage pT1b1, G2, L1, V0, R0, FIGO IB1

What would be the next steps in this case?

1. Indication for radical hysterectomy according to Wertheim with bilateral adnexectomy, bilateral pelvic SLNE. ☐
2. Cone biopsy performed, no further therapy required with R0 status ☐
3. Recommendation for simple hysterectomy or trachelectomy with SLNE ☒
4. Recommendation for simple hysterectomy or trachelectomy without SLNE ☐
