# Supplementary material for: Comparing clinical decision-making between colposcopists and large language models in cervical dysplasia management: a pilot prospective multicenter study
Source: Arch Gynecol Obstet. 2026 Jun 18;313(1):206. doi: 10.1007/s00404-026-08498-w (PMC13279379; doi:10.1007/s00404-026-08498-w)
Supplement: Supplementary file 2 — Supplementary file2 (PDF 205 KB) [file 404_2026_8498_MOESM2_ESM.pdf]

Supplemental File 2

Material and Methods

Data Analysis

Cohen's Kappa

To quantify the agreement between the two guideline authors, the inter-rater agreement score Cohen's Kappa [1] was calculated using Eq. 1.1.

$$\varkappa = \frac{p_0 - p_c}{1 - p_c} \tag{1.1}$$

p_0: proportion of questions in which the guideline authors agreed
p_c: proportion of questions for which an agreement of the guideline authors by chance is expected

Shannon Entropy

Shannon Entropy [2] was calculated to quantify the qualitative variability in therapy recommendations using Eq. 1.2. (adapted from *Wilcox* [3]). Due to the small number of datapoints within the dataset, proportion $\hat{p}_i$ was used instead of probability p_i (Eq. 1.3). The Entropy values were normalized to range [0, 1] using the maximum Entropy value as proposed, e.g. by *Wilcox* [3]. Since a uniform distribution cannot be reached, given the number of recommenders *N*=10 and the number of answer options *K*=4, instead $H_{max,actual}$ for the nearest uniform distribution was calculated and used for normalization (Eq. 1.4, adapted from [3]). $H_{max,actual}$ is constant with a value of 1.97.

$$H = -\sum_{i}^{K} p_i \cdot \log_2(p_i) \tag{1.2}$$

i: *i*-th answer option
K: total number of answer options

$$\hat{p}_i = \frac{number\ of\ recommendations\ of\ answer\ option\ i}{number\ of\ recommenders\ N} \tag{1.3}$$

$$H_{normalized} = \frac{-\sum_{i}^{K} \hat{p}_i \cdot \log_2(\hat{p}_i)}{H_{max,actual}} \tag{1.4}$$

i: *i*-th answer option
K: total number of answer options

References

1. Cohen J (1960) A Coefficient of Agreement for Nominal Scales. Educ Psychol Meas 20:37–46. https://doi.org/10.1177/001316446002000104
2. Shannon CE (1948) A Mathematical Theory of Communication. Bell System Technical Journal 27:379–423. https://doi.org/10.1002/j.1538-7305.1948.tb01338.x
3. Wilcox AR (1967) Indices of Qualitative Variation. Oak Ridge National Laboratory
