# Supplementary material for: Comparing clinical decision-making between colposcopists and large language models in cervical dysplasia management: a pilot prospective multicenter study
Source: Arch Gynecol Obstet. 2026 Jun 18;313(1):206. doi: 10.1007/s00404-026-08498-w (PMC13279379; doi:10.1007/s00404-026-08498-w)
Supplement: Supplementary file 3 — Supplementary file3 (PDF 4246 KB) [file 404_2026_8498_MOESM3_ESM.pdf]

Supplemental File 3

Suppl. Table 1: Evaluation of incorrect answers of ChatGPT-4o using the top-level error types of the error taxonomy proposed by *Roy et al.* [1]

Case Number	LLM answer	Error type	Error Evaluation
7	Question 7<br>Case: 55-year-old woman, HPV-positive, Pap IVa-p, Type III cervical lesion, no evaluable biopsy<br>Correct answer: Follow-up in 3 months, repeat colposcopy, and attempt to take a new biopsy in consultation with the dysplasia clinic<br>Rationale:<br>With Pap IVa-p and no definitive histological confirmation, further evaluation is essential. Type III TZ impairs visibility, so a repeat biopsy should be performed in the dysplasia clinic.<br>Why the others are incorrect:<br>• Routine follow-up: not justifiable if HSIL is suspected.<br>• Cervical dilation/HSK: only indicated after repeated inconclusive evaluations and in older patients.<br>• Loop excision: not indicated without histological confirmation.	Reasoning-based error	The LLM correctly states, that Pap Iva-p and unclear histology have to lead to further diagnostics, and also correctly mentions the type III transformation zone. Nevertheless, the conclusion it draws is not in concordance with the guideline as it does not prioritize the HPV infection and type III transformation zone. (Reasoning-based error)
8	Question 8<br>Case: 38-year-old woman, Pap IVa-g, histologically confirmed AIS, family planning complete<br>Correct answer: Indication for surgical treatment in the form of a hysterectomy<br>Rationale:<br>For adenocarcinoma in situ (AIS), the recommended treatment when family planning is complete is a hysterectomy. Conization is possible but does not guarantee the removal of residual lesions.<br>Why the other options are incorrect:<br>• Routine follow-up: too risky in cases of AIS.<br>• Colposcopic follow-up: not in accordance with guidelines in cases of confirmed AIS.<br>Loop excision: insufficient when family planning is complete.	Non-error type	The model presents a reasonable decision and the explanation of the chosen answer can be seen as concordant with the guideline (Non-error type).
10	Question 10<br>Case: 32-year-old woman, HPV-negative, Pap IIp, cylindrical metaplasia, plans to have children, TZ Type I<br>Correct answer: Follow-up in 3 years as part of cervical cancer screening; no further treatment required<br>Rationale:<br>Cylindrical metaplasia is a benign, physiological finding. In an HPV-negative woman with Pap IIp, no action is required before the next screening.<br>Why the other options are incorrect:<br>• Routine follow-up in 12 months: medically unnecessary.	Reasoning-based error	The model correctly states that metaplastic epithelium is a benign diagnosis, falsely concluding that for a patient tested HPV negative with Pap II-p, no action is required. It does not prioritize Pap II-p (Reasoning-based error), thus resulting in an incorrect answer that is not applicable beyond that because of the wrong screening interval regarding the patient's age (yearly screening

	• Follow-up in 6 months: not indicated with unremarkable findings.<br>• LEEP/curettage: overtreatment with normal histological findings.		would be correct for a 32 year old patient).
11	Question 11:<br>39-year-old female patient, HPV-OT positive, cytology Pap IIp, colposcopy: Type I TZ, minor changes. Histologically CIN 1.<br>Correct answer:<br>☑ Routine follow-up with a gynecologist in 12 months (cytology and HPV testing)<br>Rationale:<br>For CIN 1 in combination with Pap IIp and only minor changes on colposcopy, conservative follow-up is sufficient according to guidelines. A follow-up with HPV testing and cytology is typically performed after 12 months.<br>Why the others are incorrect:<br>• Follow-up in 6 months, dysplasia clinic: Too early and unnecessary for CIN 1 with mild findings.<br>• Cytological follow-up in 6 months: HPV testing is missing; not in accordance with guidelines.<br>• Surgical therapy (loop excision): Not indicated for CIN 1 without high-risk factors.	Reasoning-based error	The model correctly states that monitoring is indicated in this case, but then gives a vague statement ("As a rule, a check-up with HPV testing and cytology is performed after 12 months.") that does not imply the use of the guideline. (Reasoning-based error)
16	Question 16<br>Case: 61-year-old female, Pap IIIp, CIN 2, immunosuppression, HPV-positive, major changes<br>Correct answer:<br>→ Follow-up in 3 months, repeat colposcopy and biopsy in consultation with the dysplasia clinic<br>Rationale:<br>In cases of CIN 2 with immunosuppression, more frequent follow-up is indicated. The S3 guideline recommends, in the presence of risk factors (e.g., immunosuppression), a colposcopic follow-up with repeat biopsy within 3 months, particularly in cases of Pap IIIp and major changes.<br>Why the other answers are incorrect:<br>• Routine follow-up in 12 months: too late; risk increased due to immunosuppression.<br>• HPV testing and cytology in 6 months: too nonspecific; insufficient diagnostic information.<br>Loop excision immediately: overtreatment in cases of unclear persistence and without differential diagnosis.	Knowledge-based error	The model reasonably states that CIN grade 2 in combination with immunosuppression is an indication for close monitoring, but then recommends an explicitly so-called guideline-compliant therapy that is actually not supported by the guideline (Knowledge-based error).
18	Question 18<br>Case: 53-year-old female, history of LASH, HPV-positive, Pap III D2, CIN 2, p16-positive<br>Correct answer:<br>→ Indication for surgical therapy as cervical stump excision<br>Rationale:<br>In cases of CIN 2 in the cervical stump following LASH with p16 positivity (indicating	Knowledge-based error	The model incorrectly states, that conization of the cervical stump is technically limited, thus using an unsupported medical claim. (Knowledge-based Error)

	transformation), excision is indicated, as conization of the stump is technically limited.<br>Why the other answers are incorrect:<br>• Routine follow-up: insufficient for p16+ and CIN 2.<br>• Cytological follow-up or conization alone: insufficient/inappropriate for the cervical stump.		
29	Question 29<br>Correct answer:<br>Histological confirmation, PD-L1 status determination, followed by: for CPS <1, cisplatin/paclitaxel/bevacizumab; for CPS >1, cisplatin/paclitaxel/pembrolizumab<br>Rationale:<br>The patient is 85 years old, has multiple comorbidities, and has distant metastases → Stage IVB. Despite her age, according to guidelines, systemic therapy is not generally omitted but rather adapted. A PDL-1 test is also required here to determine the treatment choice.<br>Why the other options are incorrect:<br>• Wertheim surgery is contraindicated in this situation.<br>• Radiochemotherapy followed by surgery: not recommended for Stage IVB.<br>Radiation therapy alone is not sufficiently effective for distant metastases.	Reasoning-based error	The model gives correct reasoning, stating that the therapy has to be adjusted, but continues to stick with the incorrect answer option given before. (Reasoning-based error)

Suppl. Table 2: Evaluation of incorrect answers by ChatGPT-5 using the top-level error types of the error taxonomy proposed by *Roy et al.* [1]

Case Number	LLM answer	Error type	Error Evaluation
8	Question 8<br>Correct answer: Indication for surgical treatment in the form of a hysterectomy.<br>Why? In cases of histologically confirmed adenocarcinoma in situ (ACIS), the definitive treatment is hysterectomy once family planning is complete. The diagnosis is primarily confirmed via excision with endocervical curettage; hysterectomy is recommended once the patient has completed her family planning.<br>Why the other answers are incorrect:<br>• Routine follow-up in 12 months or follow-up in 3 months: Too passive for ACIS, as endocervical spread/discontinuous foci are common.<br>Loop excision: Excision is important for diagnosis and initial management; however, once family planning is complete, hysterectomy is the definitive treatment.	Non-error type	The model explains its answer reasonably and uses wording that can be found in the guideline. The chosen (incorrect) answer is reasonable according to the guideline, nevertheless it does not prioritize the excision for diagnostic purpose as indeed stated in the answer ("Excision is important for diagnosis/as a first step") that could possibly exclude the presence of an invasive carcinoma and directly chooses the more invasive and definitive option. (Non-error type)
10	Question 10<br>Correct answer: Follow-up in 3 years as part of cervical cancer screening; no further treatment required.<br>Why? Histology shows columnar epithelial metaplasia (benign), HPV negative, Pap IIp with minor changes in Zone I – there is no pathology requiring treatment. Following an unremarkable/benign evaluation, a return to routine screening is appropriate. (The IFCPC nomenclature classifies metaplasia/minor changes as normal/low-grade findings requiring no treatment.) For the organized screening program: routine screening (interval depends on age – see note below).<br>Practical note: In Germany, the routine screening interval is age-dependent: annual cytology for ages 20–34; co-testing (HPV + Pap) every 3 years starting at age 35. The core of the answer option is a return to routine screening without further treatment; for a 32-year-old, this practically means annual cytology. (The “3 years” option reflects the interval starting at age 35.)<br>Why the other answers are incorrect:<br>• Routine follow-up in 12 months (cytology and HPV): Returning to routine care is correct; an additional HPV test is not recommended for the 20–34 age group and is not medically necessary.<br>• Check-up in 6 months/repeat colposcopy: Excessive, as benign histology is present.<br>• Further surgical evaluation (LEEP/curettage): No indication with benign findings and no HPV risk.	Reasoning-based error	The model reasonably (and correctly) argues, that no therapy is indicated, but at the same time ignores the relevant information Pap II-p from the casuistic. The model correctly states, that the chosen incorrect answer does not apply to the patient case because of the wrong screening interval regarding the patient's age (3-year-screening interval for a 32-year-old patient). Nevertheless, it chooses this answer. (Reasoning-based error)

11	Question 11<br>Correct answer: Follow-up in 6 months, repeat colposcopy and biopsy, with consultation from the dysplasia clinic.<br>Why? According to the guidelines, histologically confirmed CIN 1 should be re-evaluated after 6 months. The re-evaluation refers to a colposcopic follow-up (with biopsy if necessary), not just cytology, especially if the transformation zone is clearly visible (Type I) and a lesion was initially biopsied. The risk of progression is low; therefore, a watch-and-wait approach with timely follow-up (6 months) is recommended.<br>Why the others are incorrect:<br>• Routine follow-up in 12 months (cytology + HPV) – incorrect/too late: For CIN 1, the guideline explicitly specifies a 6-month re-evaluation; annual follow-ups are only mentioned as a possibility in specific scenarios (e.g., endocervical CIN 1).<br>• Cytological follow-up in 6 months at the gynecologist – insufficient: Cytology alone without a repeat colposcopic evaluation does not meet the recommended re-evaluation criteria for confirmed CIN 1.<br>• Surgical loop excision – overtreatment: For CIN 1, excision is not primarily indicated; observation and follow-up are recommended.	Reasoning-based error	The model correctly states the reevaluation after 6 months and also uses guideline wording. Nevertheless, for re-evaluation, it chooses the more invasive diagnostics including dysplasia clinic despite re-biopsy not being indicated according to the guideline. It explains its choice using vague terms ("biopsy if applicable") and unreasonable reasoning ("not only cytology if transformation zone is clearly visible (type I)"). (reasoning-based error)
18	Question 18<br>Case: 53-year-old woman, history of LASH, CIN 2 on the cervical stump, p16-positive.<br>Correct answer: ☑ Indication for surgical treatment as cervical stump excision<br>Rationale:<br>• After hysterectomy, a cervical stump remains that may still develop CIN.<br>• In cases of CIN 2 in the cervical stump, excision of the stump is recommended, as complete removal of the lesion cannot otherwise be guaranteed.<br>• CIN 2 is considered a precancerous condition → surgical treatment is necessary, especially in cases of p16 positivity (high-risk lesion).<br>Why the other options are incorrect:<br>• “Routine follow-up” or “follow-up in 3 months” → not in line with guidelines; risk of progression too high.<br>• “Conization” → technically not possible, as there is no longer an intact cervix.	Knowledge-based error	The model correctly states that surgical therapy is necessary and also considers the p16 positivity, but then draws a conclusion that is not concordant with the guideline, arguing by using the unsupported medical claims, that extirpation is recommended. (Knowledge-based error)
20	Question 20<br>Case: 38-year-old woman, 16 weeks pregnant, Pap IVa-p, HPV 16 positive, major changes, no invasion.<br>Correct answer: ☑ Routine follow-up in 3 months with cytology and HPV testing, continued screening until delivery, then dysplasia clinic<br>Rationale:<br>• According to the guidelines for the prevention of cervical cancer (Section 15.3), no surgical	Reading comprehension error, Reasoning-based error	The model states the guideline compliant therapy for CIN2/3 and ACIS not considering that these diagnoses are not mentioned in the casuistic, thus hallucinating information (Reading comprehension error). It also mentions that invasion is not detectable, nevertheless suspicion of invasion is still relevant and

	treatment should be performed for CIN 2/3 or ACIS during pregnancy if no invasive carcinoma is detectable.<br>• Colposcopic follow-ups are performed every 3 months; repeat biopsy only if progression is suspected.<br>• Treatment no earlier than 6–8 weeks postpartum.<br>Why the others are incorrect:<br>• “HPV vaccination during pregnancy” → contraindicated.<br>• “Immediate biopsy” → necessary only if invasion is suspected.<br>• “Conization during pregnancy” → contraindicated without suspicion of invasion (risk of bleeding, preterm birth).		has to be further clarified, meaning, the model either ignored relevant information or did not draw the right conclusion from the casuistic's wording leading to an incorrect answer (Reasoning-based error).
21	Question 21<br>Case Summary:<br>44 years old, family planning complete, pT1b1 (1.5 cm, 0.8 cm depth), G2, L0, V0, Pn0, R0, FIGO IB1, HPV16+, breastfeeding.<br>Correct answer:<br>➡ Radical hysterectomy according to Wertheim, bilateral pelvic sentinel lymph node biopsy (SLNB), inclusion in the G-LACC study if applicable.<br>Rationale:<br>According to the 2022 S3 Guideline on Cervical Cancer, Chapter 8.6.2 “Standard Therapy for Invasive Cervical Cancer,” the standard of care for FIGO IB1 tumors ≤ 2 cm in patients who have completed family planning is radical hysterectomy (Wertheim-Meigs) with pelvic lymphadenectomy or SLNE.<br>The G-LACC study investigates whether minimally invasive procedures are oncologically equivalent—however, open Wertheim surgery is currently preferred because it demonstrates better survival (LACC Trial 2018).<br>Why the other options are incorrect:<br>• Similar to the SHAPE study: TLH and bilateral pelvic SLNE → The SHAPE study examines non-radical hysterectomies for tumors ≤2 cm, but has not yet been fully implemented; currently not standard.<br>• Radical hysterectomy + complete pelvic/para-aortic LNE → Para-aortic LNE only for c/pN+ or more advanced stages.<br>Primary chemoradiotherapy → Indicated only for inoperable or advanced stages (IIB and above) or in cases of contraindications to surgery.	Non-error type	This is a reasonable response generated by GPT-5 that is guideline-compliant, considering that the SHAPE study is not yet implemented within the guideline (as correctly mentioned by the LLM). (Non-error type)
29	Question 29<br>Correct answer: Histological confirmation, determination of PD-L1 status; (if appropriate) first-line systemic therapy with cisplatin (or carboplatin) + paclitaxel + bevacizumab.<br>(Among the provided answers, the first one is again the most appropriate—however, according to the guidelines, pembrolizumab should only be used as second-line therapy for	Reasoning-based error	The model correctly states the guideline-compliant therapy and correctly states, that (without relating to the given casuistic) radiotherapy as monotherapy is not sufficient in the presence of metastasis. The model also correctly mentions the casuistic facts

	PD-L1 ≥ 1. In cases of very high comorbidity or age-related risk, an adjustment to monotherapy or a purely palliative/supportive strategy should be discussed.)<br>Rationale:<br>This is also a metastatic situation. The guideline-recommended first-line treatment is Cis/Pac + Bevacizumab; in platinum-pretreated or comorbid patients, carboplatin can replace cisplatin (similar efficacy, less nephrotoxicity/neutropenia). Pembrolizumab is not the first-line standard in the current guideline, but rather second-line for PD-L1 ≥ 1. For an 85-year-old with CHD, CKD Stage II, Crohn's disease, etc., the regimen must be strictly tailored to the individual (dose, possibly a carboplatin-based regimen, or monotherapy / "best supportive care" if not eligible for treatment). Radiation therapy alone is not sufficient systemic therapy for distant metastases.<br><br>Why the other options are incorrect:<br>• Primary Wertheim procedure + metastasis excision + adjuvant radiation<br>Not standard for primary metastatic disease; systemic therapy is guideline-compliant.<br>• Primary chemoradiotherapy, followed by surgery<br>Local therapy does not address the systemic disease.<br>• Radiation therapy alone due to age/comorbidities<br>Insufficient for distant metastases; at most, used additionally for symptom management/palliative care.		(comorbidity, age) that would indicate an adjusted therapy regimen and that it has to be adjusted. Nevertheless, for its conclusion, it ignores its own reasoning and the casuistic facts that indeed are an indication for a symptom oriented / palliative setting, despite mentioning it exactly this way when refusing the correct answer option. (Reasoning-based error)
30	Question 30<br>Correct answer: Indication for radical Wertheim hysterectomy with bilateral adnexectomy and bilateral pelvic SLN dissection.<br>(In terms of content, the "bilateral adnexectomy" component is not mandatory for a 35-year-old patient without ovarian involvement; the ovaries should generally be preserved or may be fixed in place via ovariopexy. However, the key point is: For FIGO IB1 with risk factor L1, radical hysterectomy (with pelvic sentinel node staging) is the guideline-recommended standard; a simple hysterectomy would be insufficient. Fertility preservation (radical trachelectomy) is recommended only for tumors < 2 cm without risk factors.)<br>Rationale:<br>After conization: pT1b1 (FIGO IB1), 11 mm, infiltration 7 mm, L1, R0. For IB1, the guideline specifies radical hysterectomy (Piver II) with pelvic sentinel node-based staging as the standard. Fertility preservation (radical trachelectomy) is recommended only for tumors < 2 cm without risk factors; L1 is a clear risk	Knowledge-based error, Non-error type	The model correctly states that L1 is a risk factor but oversees, that L1 does not serve as single risk factor. The model builds its reasoning upon the presence of this risk factor (being a knowledge error because of incomplete understanding of the context) and thus comes to the incorrect conclusion, that fertility conservation is not possible. (Knowledge-based error) It reasons, that a simple hysterectomy is not sufficient, which indeed is indicated according to the SHAPE study, but which is not implemented in the guideline and thus cannot be used by the model. (Non-error type)

	factor. A simple hysterectomy (Piver I) is not sufficient for IB1. (Note: In premenopausal patients, consider preserving the ovaries or performing ovariopexy.)<br><br>Why the other options are incorrect:<br>• “R0 conization – no further treatment”<br>Insufficient for IB1; macroinvasive carcinoma with L1 requires definitive primary treatment.<br>• Simple hysterectomy/trachelectomy with SLNE<br>Simple hysterectomy is insufficient for IB1; Trachelectomy in patients wishing to have children would, in principle, be organ-preserving, but only in the absence of risk factors (here: L1 positive → not recommended).<br>• Simple hysterectomy/trachelectomy without SLNE<br>Both insufficiently radical (HE) and lacking necessary nodal staging — not in accordance with guidelines.		

References

1. Roy S, Khatua A, Ghoochani F, Hadler U, Nejdl W, Ganguly N. Beyond Accuracy: Investigating Error Types in GPT-4 Responses to USMLE Questions. In: Hui Yang G, Wang H, Han S, Hauff C, Zuccon G, Zhang Y, editors. SIGIR 2024: The 47th International ACM SIGIR Conference on Research and Development in Information Retrieval; 14 07 2024 18 07 2024; Washington DC USA. New York, NY, USA: ACM; 2024. p. 1073–1082. doi:10.1145/3626772.3657882.
